# End-of-life care for homeless people in shelter-based nursing care settings: A retrospective record study

**DOI:** 10.1177/0269216320940559

**Published:** 2020-07-30

**Authors:** Sophie I van Dongen, Hanna T Klop, Bregje D Onwuteaka-Philipsen, Anke JE de Veer, Marcel T Slockers, Igor R van Laere, Agnes van der Heide, Judith AC Rietjens

**Affiliations:** 1 Department of Public Health, Erasmus University Medical Center, Rotterdam, The Netherlands; 2 Department of Public and Occupational Health, Amsterdam Public Health Research Institute, Expertise Centre for Palliative Care, Amsterdam UMC, VU University Amsterdam, Amsterdam, The Netherlands; 3 Netherlands Institute for Health Services Research (NIVEL), Utrecht, The Netherlands; 4 CVD Havenzicht, Rotterdam, The Netherlands; 5 Netherlands Street Doctors Group (NSG), Amsterdam, The Netherlands

**Keywords:** Medical records, homeless persons, vulnerable populations, palliative care, epidemiology, patient transfer, social support

## Abstract

**Background:**

Homeless people experience multiple health problems and early mortality. In the Netherlands, they can get shelter-based end-of-life care, but shelters are predominantly focused on temporary accommodation and recovery.

**Aim:**

To examine the characteristics of homeless people who reside at the end-of-life in shelter-based nursing care settings and the challenges in the end-of-life care provided to them.

**Design:**

A retrospective record study using both quantitative and qualitative analysis methods.

**Setting/participants:**

Two Dutch shelter-based nursing care settings. We included 61 homeless patients who died between 2009 and 2016.

**Results:**

Most patients had somatic (98%), psychiatric (84%) and addiction problems (90%). For 75% of the patients, the end of life was recognised and documented; this occurred 0–1253 days before death. For 26%, a palliative care team was consulted in the year before death. In the three months before death, 45% had at least three transitions, mainly to hospitals. Sixty-five percent of the patients died in the shelter, 27% in a hospital and 3% in a hospice. A quarter of all patients were known to have died alone. Documented care difficulties concerned continuity of care, social and environmental safety, patient–professional communication and medical-pharmacological alleviation of suffering.

**Conclusions:**

End-of-life care for homeless persons residing in shelter-based nursing care settings is characterised and challenged by comorbidities, uncertain prognoses, complicated social circumstances and many transitions to other settings. Multilevel end-of-life care improvements, including increased interdisciplinary collaboration, are needed to reduce transitions and suffering of this vulnerable population at the end of life.

**What is already known about the topic?**

- Compared to the general population, homeless people die relatively young, with average ages of death varying between 40 and 65 years in different Western studies.
- Homeless people have disproportionally high rates of somatic and psychiatric problems, addiction, intellectual disability and other psychosocial problems, which often co-occur at the end of life.
- End-of-life care needs of homeless people are complex, among other things, because of their harsh living conditions and frequent lack of social support networks and health insurance.

**What this paper adds?**

- Although the end of life is recognised and documented for most of the homeless people who spend their final period in shelter-based nursing care settings, their end-of-life trajectories are generally hard to predict.
- Many different care disciplines are involved in shelter-based end-of-life care for homeless people, but currently, it still seems unfeasible to continuously organise and integrate end-of-life care in the shelter-based nursing care setting: 75% of the patients are transferred more than once to another institution, with almost 50% experiencing at least three such transitions.
- Two-thirds of the homeless people in shelter-based nursing care settings receive some sort of informal social support in the month before death, yet social circumstances at the end of life are often complicated and 25% of the patients are known to die alone.

**Implications for practice, theory or policy**

- End-of-life care for homeless people in shelter-based nursing care settings could be improved by more comprehensive collaboration between professionals in social care, medical specialist care, mental healthcare and palliative care.
- Structural shortages in facilities and expertise need to be addressed to improve end-of-life care options for homeless people in shelter-based nursing care settings.
- Future research might utilise local and international differences in end-of-life care models to identify successful elements of end-of-life care provision to homeless people.

## Background

People experiencing homelessness are a special, yet understudied population in the field of palliative care. Compared to the general population, they have high rates of early mortality, with average ages of death varying between 40 and 65 years in different Western population-based studies.^1–10^ Also in the Netherlands, homeless people have a substantially reduced life expectancy: a 10-year follow-up study demonstrated that the average remaining life expectancy at age 30 was 11.0 (95% confidence interval (CI): 9.1–12.9) years shorter for homeless men and 15.9 (95% CI: 10.3–21.5) years shorter for homeless women than for men and women in the general population, respectively.^11^

Besides dying at a younger age, homeless people generally also spend more years in poor health than housed individuals. Research has shown a disproportionally high prevalence of somatic and psychiatric problems, addiction, intellectual disabilities and other psychosocial issues.^5,12–15^ These problems prevail at the end of life, resulting in challenges for palliative care provision.

Indeed, various studies suggest that end-of-life care for homeless people is highly complex, among other things because of their harsh living conditions and frequent lack of social support networks and health insurance.^16–25^ However, many of these studies have been conducted in countries where the majority of homeless people die on the streets, in hostels or in acute care hospitals, such as the United States and the United Kingdom.^17,20,22–24,26^ In several countries, including the Netherlands, some social relief shelters provide 24/7 in-patient nursing care.^27^ These shelters accommodate a significant proportion of the homeless people at the end of life, but little is known about these people’s sociodemographic and health-related characteristics. In addition, as the Dutch shelter-based nursing care settings are primarily focused on temporary accommodation and recovery,^19,27^ it is unclear to what extent they are capable of providing end-of-life care. Therefore, this retrospective record study aimed to describe the characteristics of homeless people who reside at the end of life in Dutch shelter-based nursing care settings and to examine the end-of-life care provided to them as well as the documented difficulties in end-of-life care.

## Methods

### Design and setting

We performed a retrospective record study at two Dutch shelter-based nursing care settings. Both settings are social relief shelters that provide 24/7 in-patient nursing care and at least biweekly consultations with either a general practitioner specialised in street medicine (one shelter) or a municipal public health service physician (the other shelter). They have limited capacity (20 and 60 beds, respectively) and are officially intended at providing short-term care and accommodation (for a maximum of three months).^27^

### Study population

We included all persons who were known to have died (either expectedly or unexpectedly) between 2009 and 2016 and to have resided in one of the shelter-based nursing care settings for at least one night in the three months prior to death. To be admitted, people had to be diagnosed with severe somatic problems, combined with psychiatric and/or psychosocial problems. They had to be dependent on social relief and have a history of homelessness, that is, lacking housing or residing primarily in transitional housing or supervised facilities that provide temporary living accommodations (e.g. shelters).^28^ For the ease of reading, in this paper, we will refer to them as ‘homeless people’ or ‘homeless patients’.

### Data collection

Data were obtained between September 2016 and February 2017. Medical and nursing records were collected and provided by shelter staff, who verified that patients had died based on autopsy reports, death certificates or death notifications from the institutions where death had occurred.

### Measurements

To systematically extract relevant data, we developed a data extraction form.^29^ This form included pre-categorised as well as open items about (1) availability and comprehensiveness of records, (2) patients’ characteristics and diagnoses upon their last shelter admission, (3) recognition and discussion of the end of life, (4) care provision and symptoms at the end of life, (5) medical decision-making and transitions between settings at the end of life, (6) informal social contact at the end of life and sociodemographic characteristics of death, and (7) difficulties in care provision at the end of life.

We operationalised the end of life as the year prior to death.^30^ However, to get more specific information about the circumstances surrounding death, for some variables, data were only collected for the three months (i.e. symptoms, transitions between settings) or month (i.e. informal social contact) before death.

A transition was defined as a change of setting for at least one night. The end of life was considered to have been recognised and documented if the medical record included an explicit statement expressing that the patient (1) had a limited life expectancy or an unfavourable prognosis of a life-threatening disease or was in the palliative or terminal phase of life; (2) had no more curative treatment options or received palliative care; or (3) was transferred to a hospice. If the record contained more than one such statement, the first statement was used. Care difficulties were registered if the researcher identified descriptions of situations perceived to impede quality or provision of care. A palliative care team or consultant was considered to have been involved if the record included a statement describing consultation about palliation (either face-to-face or by phone, fax or email) with one or more experts of a palliative care team or service (e.g. a hospice).^31^

### Data extraction

Two researchers (S.v.D. and H.T.K.) independently pilot-tested the data extraction form on the records of five patients from both settings. They concluded that it worked well and did not require any further adjustments. Records of the remaining 51 patients were extracted and analysed by one researcher (S.v.D.); a random sample of 10 records were checked by another researcher (H.T.K.). Cases of disagreement and uncertainty were discussed and resolved with members of the project team.

### Data analysis

Data were entered in SPSS version 24.0 and Excel 2016. Open answers were categorised using both empirical approaches (i.e. categorisation guided by the data) and theoretical approaches (i.e. categorisation based on expert opinion and classification systems commonly used) approaches. Subsequently, for each of the quantitative variables, descriptive statistics were computed. Missing data were treated as a distinct category if a variable had missing data for ⩾10% of the patients.^32^ Qualitative information was analysed following the principles of thematic analysis (i.e. coded, classified into themes and discussed within the project team).^33^

### Ethical approval

The Medical Research Ethics Committee of the Amsterdam UMC (VUmc) declared that this study was exempt from formal review because it was not subject to the Medical Research Involving Human Subjects Act (registration number: 2016.308).

## Results

### Availability and comprehensiveness of medical and nursing records

Records of 61 homeless people were included. Thirty-six (59%) of these records were derived from one shelter and 25 (41%) from the other. For all but two patients (97%), both medical and nursing records were available, and results were obtained for all variables of interest.

### Patients’ characteristics and diagnoses upon their last shelter admission

The majority of homeless patients were male (85%) and either had a Dutch (56%) or Surinamese/Antillean (28%) cultural background (see Table 1). Seven percent of the patients did not have legal status in the Netherlands; a quarter did not have health insurance. Over half of them came from another social relief facility (i.e. outreach services, supportive housing/rooming-house or night shelter). Most patients had a combination of somatic (98%), psychiatric (84%) and addiction problems (90%). The duration of stay at the shelter-based nursing care setting varied between patients from 1 day to more than 10 years.

**Table 1.** Characteristics and diagnoses at the end of life among homeless people in shelter-based nursing care settings (*N* = 61).

	*N* (%)
Age in years upon last shelter admission, mean (*SD*); (min–max)	55 (10); (31–79)
Duration of last shelter stay in days, median [IQR]; (min–max)	123 [31–302]; (1–4491)
Sex: male	52 (85)
Cultural background:	
Dutch	34 (56)
Surinamese/Antillean	17 (28)
Turkish/Moroccan	2 (3)
East European	1 (2)
Other, Western	2 (3)
Other, non-Western	5 (8)
Legal residential status: no	4 (7)
Health insurance: no	15 (25)
Housing status prior to last shelter admission:	
Independent, private or public housing	3 (5)
Temporary address at friends’ or family members’ place	6 (10)
Independent, outreach services	12 (20)
Supportive housing/Rooming-house	21 (34)
Night shelter	4 (7)
Street/Sleeping rough	5 (8)
Other (e.g. detention, drug rehabilitation centre, nursing home)	10 (16)
Somatic diagnoses^a^: yes, i.e. (*more than one option possible*)	60 (98)
Cancer	30 (49)
Respiratory disease	44 (72)
Cardiovascular disease	35 (57)
Diabetes mellitus II	7 (11)
Infectious disease	31 (51)
Liver disease	19 (31)
Injury	25 (41)
Musculoskeletal disease	18 (30)
Dental problems	16 (26)
Psychiatric diagnoses^b^: yes, i.e. (*more than one option possible*)	51 (84)
Psychotic disorder	20 (33)
Depression and anxiety disorder	22 (36)
Personality disorder	12 (20)
Psycho-organic syndrome	34 (56)
Intellectual disability	13 (21)
Other (e.g. suicidal thoughts or attempts, autism spectrum disorder)	19 (31)
Addiction diagnoses (excluding tobacco)^c^: yes, i.e. (*more than one option possible*)	55 (90)
Alcohol	34 (56)
Cannabis	21 (34)
Cocaine	36 (59)
Heroin	34 (56)
Methadone	31 (51)

*SD*: standard deviation; Min: minimum; Max: maximum; IQR: interquartile range.

a No/Not in record: *N* (%) = 1 (2).

b No/Not in record: *N* (%) = 10 (16).

c No/Not in record: *N* (%) = 6 (10).

### Recognition and discussion of the end of life

For 75% of the patients, the record contained a statement indicating that the end of life was recognised (see Table 2). Patient–professional end-of-life discussions were reported for 59% of the patients.

**Table 2.** Recognition and discussion of the end of life and care provision and symptoms at the end of life among homeless patients in shelter-based nursing care settings (*N* = 61).

	*N* (%)
End of life recognised and documented in record: yes	46 (75)
End of life discussed with patient^a^: yes	36 (59)
Moment at which recognition of the end of life was first stated (number of days before death),^b^ median [IQR]; (min–max)	67 [18–170]; (0–1253)
Care discipline involved in *the year before death*: yes, i.e. (*more than one option possible*)	61 (100)
Social work	61 (100)
General practitioner care	61 (100)
Nursing care	61 (100)
Mental healthcare (e.g. addiction care, psychiatric care)	43 (71)
Medical specialist care	60 (98)
General internal medicine	36 (59)
Pulmonology	26 (43)
Surgery	20 (33)
Radiology	16 (26)
Cardiology	14 (23)
Gastroenterology	14 (23)
Neurology	12 (20)
Oncology	10 (16)
Dental surgery	9 (15)
Other (e.g. rehabilitation care, infectious diseases)	26 (43)
Dietetic care	15 (25)
Physiotherapy	20 (33)
Spiritual care	10 (16)
Volunteer services/Buddy care	2 (3)
Pedicure	13 (21)
Palliative care team or consultant	16 (26)
Symptoms in *the three months before death*^c^: yes, i.e. (*more than one option possible*)	59 (97)
Pain	55 (90)
Fatigue/Drowsiness	52 (85)
Restlessness/Confusion	44 (72)
Shortness of breath	43 (70)
Diarrhoea/Constipation	35 (57)
Nausea/Vomiting	30 (49)
Cachexia/Sarcopenia	37 (61)
Fall accidents or increased fall risk	15 (25)
Peripheral oedema	25 (41)
Ascites	6 (10)
Icterus	6 (10)
Skin problems	22 (36)

IQR: interquartile range; Min: minimum; Max: maximum.

a No/Not in record: *N* (%) = 10 (16); Not applicable: *N* (%) = 15 (25).

b *N* = 46 (i.e. patients for whom the end of life was recognised and documented in the record).

c No/Not in record: *N* (%) = 2 (3).

The moment at which recognition of the end of life was first stated ranged from almost 3.5 years before death to the day of death. The content of the statements varied as well (see examples in Box 1): whereas some statements explicitly mentioned palliative care interventions (e.g. involvement of chaplain; P50) or end-of-life decisions taken (e.g. new or updated resuscitation policies; P12 and P50), others were less explicit about care and treatment implications (e.g. P37). Furthermore, actual end-of-life trajectories could be very different from the initial expectations of care professionals, as was the case for this patient:

P09 – Last year, patient had ended up in a terminal situation, which was partly due to his frequent cocaine use. Apparently, however, he has somehow gotten out of it again. (180 Days prior to death.)

**Box 1.** Examples of statements describing recognition of the end of life in medical records of homeless patients in shelter-based nursing care settings.

P01 – Patient declared to his internist that he wants to quit chemotherapy. Oncologist: life expectancy of two months. (77 Days prior to death.) P12 – Conversation about the end of life. Patient was admitted to shelter-based nursing ward with a crack lung, chronic obstructive pulmonary disease, and heart failure. Previously, care professionals barely managed to get him off ventilator. Although patient hopes everything will still be done, his general practitioner, internist, pulmonologist and I together decided that resuscitation and hospitalisation are medically useless. (36 Days prior to death.) P37 – Current somatic problems: patient is extremely tired, feels already exhausted when waking up. Terminal renal failure, human immunodeficiency virus, heart failure, hepatitis C, chronic obstructive pulmonary disease. Patient should have had an appointment with his cardiologist and internist this month, but did not show up. (40 Days prior to death.) P50 – Incurable adenocarcinoma. Patient does not want any more invasive treatments; do not resuscitate policy, no hospital admissions unless not burdensome and with favourable prognosis for comorbid diseases. Involve a chaplain. (699 Days prior to death.)

### Care provision and symptoms at the end of life

In the year prior to death, the majority of the patients received a combination of social care (100%), nursing care (100%), general practitioner care (100%), medical specialist care (98%) and mental healthcare (70%; see Table 2). For 26% of the patients, a palliative care team or consultant was involved. The most frequently stated reasons for involving palliative care experts were pain and symptom management (e.g. medication management or palliative sedation), behavioural and psychosocial problems and care transitions (results not shown in Table 2). Symptoms reported for over 70% of the patients in the three months before death were pain (90%), fatigue/drowsiness (85%), restlessness/confusion (72%) and shortness of breath (70%). The following example shows the complexity of many palliative care consultation requests:

P36 – Please help to assess potential preferences and options to alleviate suffering of a patient with malignancy, Cushing’s syndrome, multiple drug addictions and a long history of psychiatric problems. Pain is not under control. However, this also seems to be affected by a psychiatric component, i.e. anxiety and confusion. Gradually, an unmanageable situation of refractory symptoms is arising. Patient indicated to take an overdose of methadone in case of ongoing unbearable suffering. After mentioning the consequences and the options for better supportive care, we (care professionals) could talk her out of this idea. We need to combine hospital care, primary healthcare and addiction care. (304 Days prior to death.)

### Medical decision-making and transitions between settings at the end of life

Records of 67% of the patients contained a resuscitation policy, which mostly (62% of the patients) indicated that the patient would not be resuscitated (see Table 3). For 39% of the patients, physicians had established a hospital admission policy, which predominantly (36% of the patients) concerned a non-admission decision. In the final three months before death, 77% of the patients were transferred at least once to another setting and 45% of the patients had three or more such transitions. This mainly involved transitions to acute care hospitals and intensive care units of acute care hospitals (70% and 23% of the patients, respectively, including patients with a hospital non-admission policy), and to a lesser extent to mental healthcare institutions (10%), hospices/nursing homes (8%) and detention (7%) (see Table 3). Patient–professional discussions about euthanasia were reported for 16% of the patients; in two patients, euthanasia was performed.

**Table 3.** Medical decision-making and transitions between settings at the end of life among homeless patients in shelter-based nursing care settings (*N* = 61).

	*N* (%)
Resuscitation policy documented in record: yes, i.e.	41 (67)
No resuscitation	38 (62)
Resuscitation	3 (5)
Resuscitation carried out^a^: yes	6 (10)
Hospital admission policy documented in record: yes, i.e.	24 (39)
No hospital admission	22 (36)
Hospital admission	2 (3)
Transitions between settings in *the 3 months before death*^b^: yes, i.e.	46 (77)
One transitions	9 (15)
Two transitions	10 (17)
Three or more transitions	27 (45)
Types of transitions between settings in *the three months before death*; at least one transition to (*more than one option possible*)^b^:	
Acute care hospital	42 (70)
Intensive care unit of acute care hospital	14 (23)
Mental healthcare institution	6 (10)
Hospice/Nursing home	5 (8)
Detention	4 (7)
Euthanasia discussed with patient^c^: yes	10 (16)
Euthanasia performed: yes	2 (3)

a No/Not in record: *N* (%) = 55 (90).

b *N* = 60 (the record of 1 patient did not contain sufficient information to examine transitions).

c No/Not in record: *N* (%) = 51 (84).

### Informal social contact at the end of life and sociodemographic characteristics of death

Records of 88% of the patients provided information about informal social contact in the month before death (see Table 4). Two-thirds (67%) of the patients had received some sort of social support, mainly from family (66%). Several patients had increased or even restored contact with loved ones, sometimes with the help of shelter staff. This is shown by the following extract:

P11 – Patient is single and has one little son who has been placed in custody care recently. Today, foster father came with this son to visit patient. Patient is happy about this and has started writing a little book for his son. He would like to get in touch with his family in Turkey. Possibly, we can arrange contact by emailing the town hall of his hometown.

**Table 4.** Informal social contact at the end of life and sociodemographic characteristics of death among homeless patients in shelter-based nursing care settings (*N* = 61).

	*N* (%)
Informal social contact in *the month before death:*	
Yes, i.e. with (*more than one option possible*)	41 (67)
Family/Partner	40 (66)
Friend/Acquaintance	16 (26)
No	13 (21)
Not in record	7 (12)
Cause of death^a^:	
Natural	55 (93)
Non-natural^b^	4 (7)
Place of death^c^:	
Shelter-based nursing care setting	39 (65)
Hospital	16 (27)
Hospice	2 (3)
Other: street, psychiatric hospital, detention, general practice	3 (5)
Presence of others *at the moment of death*:	
Yes, i.e. (*more than one option possible*)	26 (43)
Care professional	17 (28)
Family/Partner	11 (18)
Friend/Acquaintance	2 (3)
No	15 (25)
Not in record	20 (32)
Age in years *at the moment of death*,^b^ mean (*SD*); (min–max)	56 (9); (38–79)

*SD*: standard deviation; Min: minimum; Max: maximum.

a *N* = 59 (records of 2 patients did not contain information about the cause of death).

b Injury: *N* (%) = 2 (3); euthanasia: *N* (%) = 2 (3).

c *N* = 60 (the record of 1 patient did not contain information about the place of death).

About one-fifth of the patients did not see anyone other than care professionals or fellow patients in the month before death. Some records included an explicit statement that the patient did not want or appreciate informal social contact, like the following extract:

P51 – Patient has two children. He tried to stay in touch after divorce, but did not get any response. His mother died and patient does not know whether his father is still alive. His sorrow about this has faded. He has no wish to get in contact with his family.

For both patients with and without informal social contact at the end of life, social circumstances were often pictured as complicated. This sometimes invoked feelings of loneliness and regret:

P60 – Patient feels lonely. On some days, he gets little attention from staff. [. . .] Today, he used the following words: ‘taken the wrong path in life’, ‘having disappointed loved ones’, ‘becoming increasingly aware that I am really all alone now’.

Table 4 shows that patients died at the average (*SD*) age of 56 (9) years. Except for 7% who died from euthanasia (3%) or due to injury (3%), almost all patients (93%) died from a natural cause. Most patients died in the shelter (65%), others in a hospital (27%) or a hospice (3%). According to 43% of the records, patients died in the presence of someone else, mostly a care professional (28%). A quarter (25%) of the patients were known to have died alone.

### Difficulties in care provision to homeless people at the end of life

Examples of documented difficulties in end-of-life care are displayed in Table 5. A recurrent issue concerned the continuity of care, which was considered to be impeded by insufficient and fragmented facilities and expertise, but also by inadequate coordination of tasks and responsibilities between care providers (P05, P39, P48) and gaps in care policies and legislations for certain subgroups, such as uninsured (P38) and psychiatric patients (P01). In addition, records contained frequent accounts of social and environmental safety problems, such as rude (P04), unhygienic (P31) and hazardous behaviours (P10, P37). Another challenge to end-of-life care provision was constituted by patient–professional communication difficulties, which were mostly attributed to characteristics of the homeless population, such as language barriers (P39), somatic functional impairments (P42) and psychiatric and behavioural problems, including care denial (P50) and lack of openness towards care professionals (P36). Finally, many records included remarks expressing persistent medical-pharmacological difficulties to alleviate suffering of homeless patients at the end of life (P11).

**Table 5.** Documented difficulties in end-of-life care provision to homeless patients in shelter-based nursing care settings.

1. Discontinuity of care due to:
• Insufficient facilities, fragmented expertise and inadequate coordination between care providers P05 – Letter to other care organisations: Patient currently lives in a small shelter-based nursing care setting, which is insufficiently equipped with the required nursing attributes [. . .] There have been long-standing placement issues with this patient: other care facilities refused to admit him because of waiting lists, unmet age requirements (i.e. patient is too young for a nursing home) or inability to handle his behaviour. P39 – Patient is back from the hospital! [. . .] Fellow patients warned us (shelter staff) that he had fallen and was lying in front of the outside door. We had not been informed about his discharge! It is unclear to us how his medication has changed. P48 – Again, patient has been hospitalised because of his heavy dyspnoea. The hospital will probably again discharge patient to us, but we (shelter staff) consider ourselves no longer able to provide responsible care. It would be most advisable to admit this patient to a nursing home. The hospital’s transfer department can arrange this much quicker than we can, but still sees no reason for referral.
• Gaps in specific care policies and legislations P01 – The court order for compulsory admission to a psychiatric hospital was not authorised by court yesterday [. . .] Care professionals are put up against the wall; psychiatric assessment is urgently needed. Patient’s choices seem to result from psychosis and depression and, moreover, cause exacerbations of these conditions. According to his oncologist, patient’s denial of chemotherapy is not understandable. P38 – Cross-border issues regarding residence permit and health insurance have resulted in uncertainty about financial reimbursement of cancer treatment, treatment delay and placement difficulties.
2. Difficulties with social and environmental safety
P04 – Patient can come across quite commanding and seems to direct this behaviour at one nurse per shift. He frequently uses the bed alarm for unclear reasons and called the police three times today. P10 – Patient’s own safety and safety of others are at risk because he persistently smokes in combination with oxygen, running the risk of being burnt alive. Additional safety measures are required: patient should bring his base pipe to the guard and visitors who facilitate drug use should be barred. P31 – Patient is hard to handle: he refuses food and care, is apathetic and angry, [. . .], makes a mess and fouls everything with sputum. He has been prohibited access to the nearby shopping mall by means of an exclusion order. P37 – Within the team (of shelter staff) there is a strong suspicion that patient deals hard drugs to fellow patients. Also, she lends fellow patients her scoot mobile in exchange for cocaine.
3. Patient–professional communication difficulties due to:
• Language barriers P39 –It is hard to communicate with this patient because of his lack of understanding of the Dutch language combined with psychiatry, behavioural problems and intellectual disability.
• Somatic functional impairments P42 – Patient is deformed due to a resection of his jaw and tongue. [. . .] Because of his speech impairment, his needs are unclear. Patient suffers from hypersalivation, which causes feelings of shame and isolation.
• Psychosocial and behavioural problems P36 – It is difficult to monitor patient’s drug use. Again, non-prescribed drugs were found in her room. She says that she does not use them. [. . .]. Because of risks of overdosing, I (general practitioner) asked her to be honest about it. P50 – Patient denies palliative treatment [. . .] Since a week, he has shown alarming signals. However, shelter staff cannot reach him and he does not allow any check-ups.
4. Medical-pharmacological difficulties with the alleviation of suffering
P11 – Since a year already, it has been very difficult to alleviate suffering of this patient. His pain is unbearable. He does not want to swallow and agitates against the pain. [. . .] We (shelter staff) see a man who has become desperate because of the pain [. . .] We will soon run out of our own stock of pain medications.

## Discussion

### Summary of findings

Our study confirms previous findings that compared to the general population, homeless people die younger^1–11^ and have complex comorbidities and a high symptom burden at the end of life.^19–22,24,34–37^ Although the end of life was recognised for three-quarters of the homeless persons in our study, it was difficult to specifically predict prognoses and identify palliative care needs: whereas some patients revived prodigiously, others deteriorated rapidly once admitted to the shelter-based nursing care setting. This finding corroborates qualitative studies indicating that healthcare professionals experience end-of-life trajectories of homeless people to be especially capricious.^17–19,21,22^

In the year prior to death, almost all patients received care from multiple social work, medical specialist and mental healthcare services. For a quarter of them, a palliative care team was consulted, which is twice the proportion observed in the general Dutch population.^31^ Almost two-thirds died in the often familiar shelter-based nursing care setting. Nevertheless, in most cases, it seemed unfeasible to continuously organise and integrate end-of-life care in the shelters: 75% of the patients in our study were transferred more than once to another institution, with almost 50% experiencing at least three such transitions. This is a lot compared to the general Dutch people, who mostly experience no more than one transition in the three months before death.^38^ Moreover, these figures largely outnumber estimates obtained in other vulnerable populations.^39–41^ Among institutionalised people with dementia, for example, less than 10% had multiple transitions in the three months before death.^39,40^

Most records contained multiple explicit examples of discontinuity of care, social and environmental safety issues, complex communication and medical-pharmacological issues. Partially, these difficulties are inherent to the complex problems of the population. Yet, they may also be attributed to external, systemic factors. For example, unwanted hospital admissions and extended stay in the not always sufficiently equipped shelters could be explained by a lack of specialised, long-term available end-of-life care facilities for homeless people and policies hampering their placement in regular care facilities. Also, these placement issues may have deeper causes, such as a tendency among professionals in medical disciplines to shift responsibility to other care disciplines (e.g. social care) when confronted with serious psychiatric and psychosocial symptoms.^35,42,43^ Together with statements about, for instance, reimbursement issues for uninsured patients and insufficient coordination between care providers, these results elucidate pressing and ubiquitous issues of uncertainty, confusion and conflicting ideas regarding roles and responsibilities in end-of-life care for homeless people.

Although we observed some sort of informal social support among two-thirds of this shelter population, consistent with other studies,^19,34,36,43–46^ social circumstances at the end of life were often described as complicated. In addition, 25% of the patients were known to have died alone, and in reality, this percentage is probably higher, as information about the presence of others at the moment of death was unavailable for most of the patients who died in the hospital. Previous studies among homeless people have pointed out unmet needs for personal attention, understanding and family-like relationships as well as a common fear of dying alone.^22,23,36,46–50^ Hence, carefully assessing social networks and needs of homeless people is important to anticipate and reduce emotional suffering at the end of life.^51^

### Implications for practice, research and policy-making

Our findings suggest that continuity of end-of-life care for homeless people at the end of life could be improved by more comprehensive collaboration between the various care disciplines involved. The current difficulties in continuity of end-of-life care and the complex problems of the population highlight the challenges, but also the importance of individualised advance care planning.^52,53^ Furthermore, system-level changes in organisation of end-of-life care for homeless people, which take into account uncertain prognoses, are required to address structural shortages in expertise and facilities and increase end-of-life care options in shelter-based nursing care settings.^54,55^ For patients with unmet social support needs, volunteer or buddy support might be a valuable alternative, which could possibly also reduce the strain on care professionals.^24,50,56^ In research and policy-making, it is important to identify needs and self-management strategies of homeless people themselves, including those who do not seek professional care.^57^ Future studies might utilise local and international differences in care models to draw comparisons and identify successful elements of end-of-life care provision to homeless people.

### Strengths and limitations

To our knowledge, this is the first European study that provides a thorough overview of shelter-based care for homeless people at the end of life. While most studies have used cross-sectional data from interviews and focus groups, we examined real-world medical and nursing record data that were documented during the full end-of-life period. We included two of the largest shelter-based nursing care settings in the Netherlands. Still, generalisability of our results remains limited to homeless people who use such facilities. Compared to two previous, North American studies on shelter-based end-of-life care for homeless people, our study included more patients and adds quantitative findings on the number of care transitions in the months before death.^34,36^ Unfortunately, however, data collection was confined to shelter records and therefore only included information about care provision elsewhere if communicated to the shelters and put in the record. Also, data might have been prone to other types of recording bias, which may, for example, have occurred due to changes in documentation over time.^29,32^ Nevertheless, records seemed rather complete with respect to most of the variables of interest.

## Conclusion

This retrospective record study shows that at the end of life, homeless people have multiple somatic, psychiatric, addiction and social problems, for which those residing in shelter-based nursing care settings receive care from a variety of healthcare and social care disciplines. Yet, their end-of-life trajectories are uncertain and end-of-life care is fragmented, with transitions to other institutions being rather the rule than the exception. Overall, our findings paint a worrisome picture of acute and structural shortages in capacity to serve this vulnerable population at the end of life. Multilevel end-of-life care improvements, including increased interdisciplinary collaboration and more palliative care facilities and expertise within shelter-based nursing care settings, are needed to reduce unwanted transitions and suffering among homeless people at the end of life.
